# The Bacterial and Viral Agents of BRDC: Immune Evasion and Vaccine Developments

**DOI:** 10.3390/vaccines9040337

**Published:** 2021-04-01

**Authors:** Rachael Lynda Bell, Hannah Louise Turkington, Sara Louise Cosby

**Affiliations:** 1 Veterinary Sciences Division, Agri-Food and Biosciences Institute, Stormont, Belfast, BT4 3SD Northern, Ireland; hannah.turkington@afbini.gov.uk (H.L.T.); Louise.Cosby@afbini.gov.uk (S.L.C.); 2 Centre for Experimental Medicine, School of Medicine, Dentistry and Biomedical Sciences, Queen’s University Belfast, BT9 7BL Northern, Ireland

**Keywords:** bovine respiratory disease complex (BRDC), immunity, immune evasion, respiratory viruses, bacterial agents, vaccine development

## Abstract

Bovine respiratory disease complex (BRDC) is a multifactorial disease of cattle which presents as bacterial and viral pneumonia. The causative agents of BRDC work in synergy to suppress the host immune response and increase the colonisation of the lower respiratory tracts by pathogenic bacteria. Environmental stress and/or viral infection predispose cattle to secondary bacterial infections via suppression of key innate and adaptive immune mechanisms. This allows bacteria to descend the respiratory tract unchallenged. BRDC is the costliest disease among feedlot cattle, and whilst vaccines exist for individual pathogens, there is still a lack of evidence for the efficacy of these vaccines and uncertainty surrounding the optimum timing of delivery. This review outlines the immunosuppressive actions of the individual pathogens involved in BRDC and highlights the key issues in the development of vaccinations against them.

## 1. Introduction

BRDC is a term used to describe severe respiratory disease in cattle and is sometimes referred to as shipping fever due to the increased risk of infection and transmission during cattle transportation [1]. BRDC is a multifactorial disease caused by both bacterial and viral infections, with high rates of re-infection [2]. BRDC is the costliest disease in the beef industry and is the biggest cause of mortality in calves aged one to five months in Ireland, accounting for between 30 and 34% of deaths in this age group [3]. A recent study estimated costs up to USD 42.15 per affected calf [4]. BRDC is also potentially responsible for up to 70% morbidity and mortality rates in US feedlot cattle [5,6]. The risk of infection and the severity of disease is determined by the infectious agents involved, their immunogenicity, genetics and microflora of the host and external environmental factors. The infectious agents involved in BRDC are opportunistic and often enhanced by stressors such as weaning, overcrowding, mycotoxins from food contamination, along with fluctuations in temperature, humidity, air, lighting, and sound. These factors can induce a transient immunosuppressive state which allows for colonisation of pathogenic bacteria and virus replication [7]. Whilst there are preventative vaccines and antibiotic treatments available against several common BRDC agents, the specific pathogens involved in individual cases of BRDC are often unknown [1], meaning prioritising a vaccination regime is difficult.

BRDC has been thoroughly reviewed previously; the immune response has been reviewed in detail [2,8], with the developments of the last decade outlined by McGill & Sacco in 2020 [9]. The exhibition of clinical signs, times of shedding, and seroconversion of each pathogen has also been outlined by Grissett et al. [10]. This review however summarises the mechanisms of immune evasion reported for each major BRDC pathogen and the current issues in the development of effective vaccines. It is worth noting however that several additional pathogens have been implicated in the development of BRDC (for example adenovirus [11], coronavirus [12,13], influenza D [13], *Mycoplasma bovis* [14] and *Trueperella pyogenes* [14]), although these will not be discussed in detail in this review. 

## 2. BRDC Pathogenesis 

Many of the bacterial agents that are associated with BRDC are present in bovine nasal passages, without illness, the most common being *Mycoplasmas and Mannheimia haemolytica* [15]. In healthy cattle, there exists a delicate balance between these potentially pathogenic bacteria and the commensal microflora of the upper respiratory tract (URT). Key immune mechanisms, such as mucus production and ciliated epithelial movement, actively prevent the colonisation of pathogens in the lower respiratory tract (LRT). In BRDC, there is a shift in this homeostatic balance in the URT which results in colonisation of the LRT.

Once BRDC infections reach the LRT, they are often persistent and difficult to resolve due to the unique immunosuppressive and immune avoidant mechanisms exhibited by each agent. As BRDC is a multifactorial disease, co-infections work in feedback loops to enhance viral and bacterial replication, adherence, toxicity, and persistence. This is a major challenge for vaccination development, as discussed in the second half of this review. The development of safe and effective vaccines depends on the understanding of how these infections effect each stage of the immune response, outlined herein.

## 3. Cellular Response

### 3.1. Epithelial Cells

**Figure 1:**
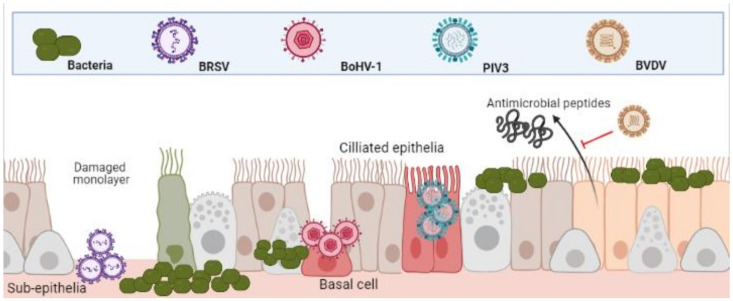
Differential infection of upper and lower respiratory epithelia and subsequent bacterial colonisation. Viral infection leads to inhibition of function and cell death. This both increases bacterial adherence to, and colonisation, of the lower respiratory tracts. (Created in BioRender.com, accessed on 24 March 2021).

The epithelia of the UTR are the first line of defence against respiratory and bacterial pathogens and therefore, often the initial sites of infection. Indeed, this is true for Bovine Respiratory Syncytial Virus (BRSV), Parainfluenza Virus 3 (PIV3) and *Alphaherpesvirus* Bovine Herpes Virus-1 (BoHV-1). BRSV initially infects both ciliated bronchial epithelia and type II pneumocytes of the respiratory mucosa [16,17]. PIV3 readily infects the respiratory ciliated epithelium [18,19]. BoHV-1 first infects mucosal epithelia of both respiratory and/or genital tracts [20,21]. Although these agents all contribute to BRDC, the mechanisms and cell type in which they initiate infection in the URT differ (depicted in Figure 1) [22]. 

It has been demonstrated that whilst PIV3 directly infects apical ciliated epithelium, these cells were resistant to BoHV-1 infection, which preferentially targeted basal epithelium of injured monolayers. In the same study, BRSV infected only the sub-epithelia [22]. The same patterns of infection were shown in a caprine lung slice model using BRSV, PIV3 and BoHV-1 [23]. Once infected, the physical defenses of epithelia to bacteria are weakened. BRSV infected epithelia in the bronchus and lung are significantly more susceptible to *Pasteurella multocida* infection [24]. BoHV-1 infected bronchial epithelium was shown to not only increase recruitment and activation of neutrophils to sites of infection but increase their susceptibility to *M. haemolytica* infection and subsequent cell death [25]. As well as physical barriers of defence, bovine epithelial cells mediate antimicrobial activity via beta-defensin secretions such as lingual antimicrobial peptide (LAP) and tracheal antimicrobial peptide (TAP) which is interrupted by the agents of BRDC [8]. For example, *Pestivirus* Bovine Viral Diarrhoea Virus (BVDV) infection has proven to decrease TAP and LAP expression after exposure to bacterial toxin lipopolysaccharide (LPS) [26,27]. This predisposes animals to bacterial infections and highlights the synergies of co-infections. 

It is important to note that although BRDC is primarily a respiratory disorder, its agents are not solely pathogens of pulmonary tissue. For example, BoHV-1 infection results in not only respiratory disorders but also conjunctivitis, and genital disorders [28]. BVDV, another prominent BRDC agent, also presents clinically in multiple organs including those of the respiratory, gastrointestinal and reproductive systems [29,30,31]. *Histophilis somni* is not exclusively a pulmonary pathogen and it is common for more invasive, systemic infections to take hold in the weeks and months following initial exposure [14,32]. *H. somni* expresses an immunoglobulin binding surface protein which induces endothelial contractions, allowing for the bacteria to enter the blood stream [33]. Subsequently, infections have been found in the reproductive tract that lead to abortion. *H. somni* has also been associated with sudden death due to cardiac complications [34]. This multi-organ infection is largely due to the ability of the pathogenic agents to infect and replicate in not just epithelia, but in key leucocytes of the innate and adaptive immune response.

### 3.2. Neutrophils, Monocytes and Macrophages

Neutrophils are the first and most rapidly recruited cells to the site of infection, via chemokine signalling from damaged epithelia [35]. BoHV-1 infected ciliated and mucosal epithelia undergo apoptosis after rapidly producing inflammatory cytokines such as IL-8 [22]. This adds to tissue damage via neutrophil recruitment and subsequent degranulation and protease release [25]. Neutrophils were identified as a key target for BVDV immunosuppression. One study showed impaired phagocytosis, increased cellular toxicity, cytochrome-C reduction, iodination, oxidant production, and cytoplasmic calcium flux in neutrophils from cattle persistently infected with BVDV, compared with non-infected controls [36]. BRSV is known to evade the killing mechanisms of neutrophils [37]. Neutrophil traps (NETs) function to trap pathogens for immune clearance but are ineffective during BRSV infection and have been implicated in airway occlusion [37]. *M. haemolytica* also contributes heavily to the fibrosis of the lung during BRDC by inducing the release of neutrophil chemoattractant IL-8 [38,39]. The detrimental nature of neutrophil recruitment was demonstrated when infection with *M. haemolytica* in a neutrophil depleted model showed decreased lung pathology and reduced inflammatory cytokine release [40].

Macrophages are the most numerous immune cells in the healthy lung, with systemic monocytes being the first cells recruited during infection after neutrophils [41]. These cells play an important role in the pathogenesis of many agents of BRDC. Monocytes function as the main carrier of infection to other leukocytes for the most prominent viral agent of BRDC, BoHV-1 [21]. Like BoHV-1, BVDV readily infects macrophages and monocytes. Specifically, it invades and replicates in alveolar macrophages, impairing function and destroying these key immune cells [42]. In vivo infection of monocytes with BVDV significantly affected their ability to act as antigen presenting cells, resulting in a poor CD4+ T-cell memory response [43]. The phagocytic killing ability of both alveolar and infiltrating macrophages is inhibited by BVDV infections, seen also with PIV3 and BRSV infections [44,45,46]. Additionally, PIV3 infected alveolar macrophages are known to exhibit contact inhibition to surrounding lymphocytes [47,48,49].

**Figure 2:**
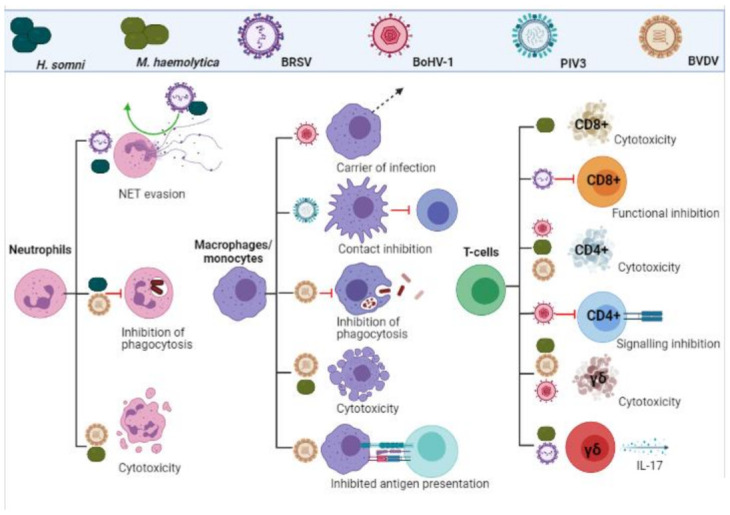
Leukocyte evasion and inhibition by BRDC pathogens. Viral and bacterial infections inhibit functionality, impact signalling and induce cytotoxicity in cells of the adaptive and innate immune response. (Created in BioRender.com, accessed on 24 March 2021).

Many of the immunosuppressive effects seen with BRDC infections are likely due to a decrease in overall white cell and platelet counts. Numerous studies have demonstrated this phenomenon in BVDV [50,51,52,53,54] and BoHV-1 [55] infections. A key strategy of *M. haemolytica* is the production of a leukocyte specific exotoxin, termed leukotoxin (LKT). This is a pore forming toxin which binds to leukocyte specific β integrin’s, resulting in inhibition of function, apoptosis and necrosis of neutrophils, macrophages and all other leukocyte subtypes [56]. The suppression of leukocyte function play a large role in pathogenesis of *H. somni* infection [57]. As well as inducing apoptosis [58], *H. somni* inhibits the production of superoxide anion by both alveolar macrophages and neutrophils [59]. As with *M. haemolytica*, there is an influx of neutrophils and NET formation upon initial infection, although infections persist [60]. An in vitro study using bovine neutrophils showed that any material phagocytosed is not destroyed when *H. somni* is present [61]. The inhibition of both macrophage and neutrophil responses are summarized in Figure 2. 

### 3.3. T-Cells

As previously mentioned, BRDC infections exhibit leukopenia, which accounts for much of the immunosuppression. It is also well documented that BRDC infections mount a poor memory response, with persistent infection and high re-infection rates [2]. Specifically, immune memory is dependent on successful signalling from innate immune cells to the adaptive immune counterparts, arguably the most important being CD8+ and CD4+ T-cells. Cytotoxic CD8+ and helper/regulatory CD4+ T-cells are essential for bacterial and viral clearance and rapid response initiation upon re-infection [62]. BRSV is known to induce cytotoxic CD8+ T-cell inhibition [16,63]. CD8+ cells are vital for the destruction of infected cells and when depleted, correlate with disease severity [16,63]. CD4+ T-cells are known to be susceptible to BoHV-1 during acute infection, resulting in cell death [64,65] and inhibition of CD4+ signalling and regulatory functions [65]. In a model of BoHV-1 infection, both populations of CD8+ and CD4+ cells were significantly deleted throughout 14 days of infection. Furthermore, this depletion was amplified by pre-existing subclinical BVDV infection, which was particularly prominent in CD8+ populations [66]. 

γδ T-cells are a small subset of T-cells in humans, although make up for 60% of circulating lymphocytes in young claves [67]. These cells are thought to play a role in early pathogen detection and recognition of viral infection [68]. There is evidence for a dual role in the innate and adaptive response to BRSV infection by γδ T-cells [69]. These cells are also known to be depleted during BVDV and BoHV-1 co-infection, to a lesser extent than CD4+/CD8+ cells [66]. This phenomenon however is not seen with BRSV infections [70]. It has been suggested that γδ T-cells play a role in viral infection, specifically RSV, via IL-17 production [71], the role of which remains controversial, both acting protective and pathogenic [72]. Indeed, one study showed that calves infected with BRSV first mounted a strong IL-17 response, mediated by CD4+ and γδ T-cells, and that co-infection with *M. haemolytica* exacerbates this IL-17 produced primarily through γδ T-cells. The beneficial or detrimental nature of this response remains unclear [73].

The inhibition of T-cell responses are summarized in Figure 2.

## 4. Cell Signalling

### 4.1. MHC Signalling

Effective synergy between innate and immune responses relies heavily on antigen presenting cells such as macrophages and dendritic cells. These cells phagocytose invading pathogens and present specific antigens via MHC class I and II proteins, along with the transporter associated with antigen processing (TAAP) [74,75,76]. BoHV-1 actively disrupts this communication by infecting and downregulating the expression of signalling molecules within lymphocytes. BoHV-1 infection is known to inhibit the translocation of internalised MHC class I receptor to the surface of infected cells [64]. MHC receptor expression is also intrinsically downregulated [77]. Newly synthesised MHC receptors are found in abundance in the endoplasmic reticulum of BoHV-1 infected cells, suggesting that interference with TAAP-dependent transport mechanisms is responsible for the lack of lymphocyte activation during infection [64,78]. Additionally, the number of cells expressing MHC class II was decreased up to 50% during infection with a non-cytopathic laboratory strain of BVDV [79]. In a mouse model of HRSV, evidence was shown to suggest that CD8+ responses are limited to do a lack of recognition of specific viral proteins due to interference in T-cell receptor medicated signalling, which normally are activated by MHC antigen presentation. This may account for the poor CD8+ proliferative response seen with HRSV and BRSV infections [80,81].

**Figure 3:**
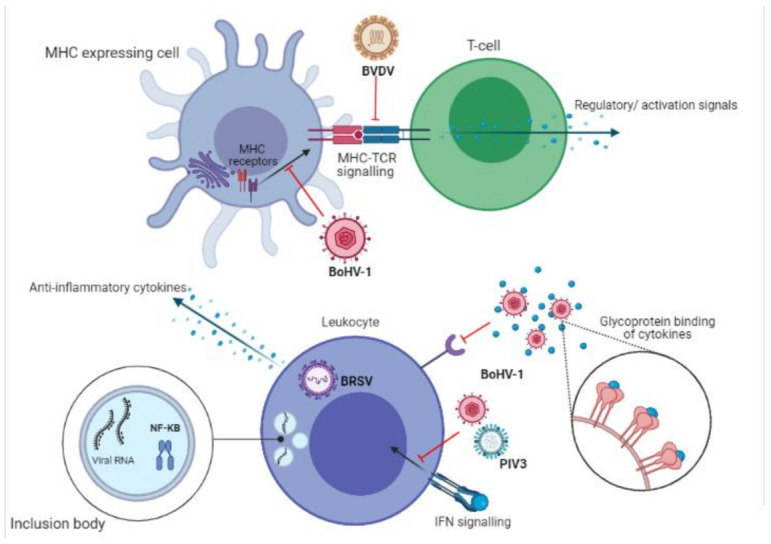
Inter- and intra-cell signalling dysregulation induced by bacterial and viral infections. Interferences with MHC protein expression and cytokine regulation result in limited anti-viral and bacterial immune responses and poor immune memory production. (Created in BioRender.com, accessed on 24 March 2021).

The inhibition of MHC signalling is summarized in Figure 3.

### 4.2. Cytokines and Chemokines 

Cytokines and chemokines are the chemical messengers which initiate the innate and link the adaptive immune responses. Dysregulation of the homeostatic balance between stimulatory and inhibitory cytokines plays a key role in the pathogenesis of viral and bacterial infections, including those of BRDC [2,9]. Increased secretion of anti-inflammatory cytokines from BRSV infected macrophages amplifies immune evasion, by dampening the initial cellular response mounted [82]. *M. haemolytica* infected macrophages were also shown to secret increased IL-10, an inhibitor of MHC II antigens, co-stimulatory molecules on macrophages and Th1 inflammatory cytokine expression [83]. Chemokine and cytokine interferences increase the immunosuppressive actions of BoHV-1 infection, further inhibiting cell mediated immunity. For example, decreases in proliferative lymphocyte responses may be due to the downregulation of IL-2 receptors on the surface of lymphocytes [84]. Direct interference occurs via BoHV-1 glycoprotein G (Gg), which binds chemokines and blocks receptor interaction [85,86]. 

Classic anti-viral interferon gamma (IFNγ) responses are also disrupted, specifically by BoHV-1 infection [87]. IFNγ is vital to both the innate and adaptive immune response. Among its many antiviral functions is the upregulation of crucial signalling peptide MHC II [88,89]. BoHV-1 viral protein VP8 interferes with host cell signalling by inhibiting the translocation of activated IFN receptors to the nucleus [90]. Signal transducer and activator of transcription 1 (STAT1), which is bound to the IFNγ receptor, would normally induce a second wave of IFNγ production, a key response absent in BoHV-1 infections [90]. PIV3 was shown to inhibit the signalling of other important antiviral interferon, Interferon β (IFNβ) [91]. IFNβ is responsible for the expression of costimulatory molecules on dendritic cells, mediation of CD8+ responses and antigen presentation via MHC I [92].

Recently, a new mechanism of immune evasion was discovered, by both human and bovine RSV, in which these viruses replicate in cells whilst blocking intra cell signalling [93]. RSV uses inclusion bodies which allow for viral RNA replication whilst simultaneously capturing transcription factor NK-KB, inhibiting nuclear translocation of this vital signalling molecule [93]. 

The inhibition of cytokine signalling is summarized in Figure 3.

### 4.3. Inflammasomes

The multifactorial nature of BRDC suggests the involvement of the inflammasome in its pathogenesis. The inflammasome is a multiprotein activating platform of inflammatory caspase-1 and inflammatory cytokines IL-18 and IL-1β [94]. Most inflammasomes are activated by an initial pathogen associated molecular pattern (PAMP) and a subsequent danger associated molecular pattern (DAMP) via pattern recognition receptor (PRR) signalling [95]. Indeed, inflammasome activation was seen with BoHV-1 infection in bovine kidney cells, but its role in pathogenesis remains unclear [96]. The NLRP3 inflammasome is also a known contributor to HRSV pathogenesis [97] and increased IL-18 expression levels have been seen in BRSV infection models [98,99]. A vital function of the inflammasome is regulation of the Th-1 and Th-2 adaptive immune responses [100,101] which are known to be altered in RSV infection, favouring immune suppression [102,103]. Additionally, the SH protein of BRSV has been proven to play a role in IL-1β expression. Infection models without the SH protein show increased IL-1β expression compared to wild type virus [99]. IL-1β is known to propagate adaptive immune response such as Th-2/Th-1 [104,105] and its regulation by the BRSV SH protein may be how the virus limits the immune response.

## 5. Viral Latency and Immune Tolerance

BoHV-1 owes its high virulence and persistence to a few key mechanisms including lifelong latency. BoHV-1 sustains latency in the neurons of the peripheral nervous system [106]. This means infections are often persistent and arise again in times of stress [107]. On initial infection, cattle mount a robust innate immune response, but viral replication and spread occurs despite this [108,109]. Stress mediates BoHV-1 infection by directly activating key viral promoters, inducing increased virus replication, and indirectly enhancing spread via immunosuppression [110,111,112].

**Figure 4:**
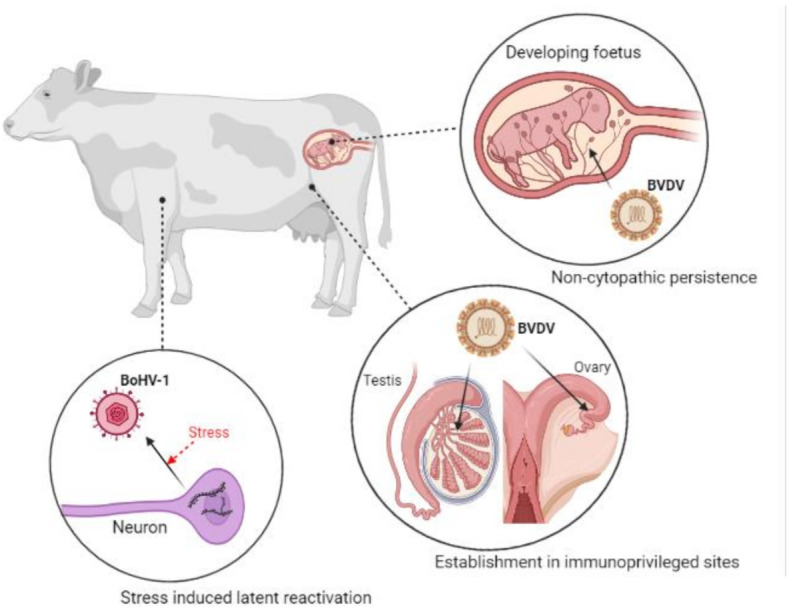
Viral reactivation from latency and establishment in sites of immune tolerance by BoHV-1 and BVDV, respectively. BoHV-1 is commonly a lifelong infection, arising in times of stress. BVDV maintains persistent infections within herds due to non-cytopathic spread from cow to foetus. (Created in BioRender.com, accessed on 24 March 2021).

BVDV is also commonly a chronic, persistent infection. It achieves this by establishing itself in immuno-privileged sites such as the ovaries and testes [113]. It has been demonstrated that prolonged infection induces a strong neutralising antibody response [113]. Despite this, BVDV persists as a non-cytopathic infection in the developing bovine foetus, successfully maintaining infections within herds [114]. Initial persistent infections maintain a state of immune tolerance in cattle and constitutively shed virus into their environment. These mechanisms are depicted in Figure 4. 

## 6. Genetic Predisposition 

For HRSV, it is unknown why some infants develop infections and others do not, a phenomenon also seen with BRSV infected calves. Multiple predisposing environmental factors have been identified which contribute to BRDC development, but these are not present in every case and cannot be used reliably to predict cases [7,115]. Genetic polymorphisms may act as diagnostic biomarkers of susceptibility to infection with HRSV [116,117] and BRSV [118,119]. 

In humans, bactericidal surfactant protein SP- A and D polymorphisms are known to increase RSV disease severity and infection [120,121]. These surfactant proteins contribute to early innate defence mechanisms by binding to mannose residues on microbes which are detected and phagocytosed by tissue resident macrophages [122]. Absence of these early defences increases colonisation of the UTR and increases subsequent infection of the LTR. 

In 2014, 116 genomic loci were identified which were associated with BRDC susceptibility [123]. A recent study identified multiple open chromatin regions in the bronchial lymph nodes of calves infected with BRSV [119]. These regions were found to be involved in Th1 and Th2 pathways, pathogen recognition and the anti-viral response [119]. Likely to be involved in the regulatory response of gene transcription induced by BRSV infection, these regions may contain genetic variations which may confer resistance to BRDC. Further characterization and investigation may provide a useful tool for genomic selection to decrease BRDC prevalence within herds [118,119].

## 7. Current Vaccines

Viral vaccines contain either modified-live virus (MLV) or inactivated virus [124,125]. MLV vaccines are known to induce robust humoral and cell-mediated immunity, and can do so with a single dose [126,127,128,129]. In contrast, inactivated vaccines prime the humoral immune response but are poor at inducing cell-mediated immunity [130]. Additionally, inactivated vaccines require a booster dose in order to achieve protection [131]. Evidence from multiple studies suggests that MLV vaccines are more effective in preventing BRDC than their inactivated vaccine equivalents [125].

Between 2011 and 2017, there were 12 multi- or mono-valent vaccines commercially available for the prevention of BRDC in the UK [132] (see Table 1). These include two monovalent vaccines against *M. haemolytica* (Bovalto Pastobov and Rispoval Pasteurella) and one monovalent vaccine against BRSV (Rispoval RS). Multivalent viral vaccines offer protection against varying combinations of BoHV-1, BRSV, PIV3 and BVDV (Bovalto Respi Intranasal, Imuresp RP, Rispoval 3, Rispoval 4 and Rispoval RS + PI3 Intranasal). A bivalent bacterial vaccine is available against *M. haemolytica* and *H. somni* (Hiprabovis SOMNI/Lkt) and three combination vaccines are available for *M. haemolytica* plus either; BRSV and PIV3 (Bovalto Respi 3 and Bovilis Bovipast RSP), or BRSV, PIV3 and BVDV (Bovalto Respi 4). Additionally, there are 7 BoHV-1 specific monovalent vaccines on the market [132].

In the UK, the percentage of calves vaccinated in their first year of life with the above vaccines for prevention of pneumonia unfortunately remains relatively low. Vaccine uptake for the prevention of pneumonia was estimated at only 29% in 2011 but did rise to 38% in 2017 [132]. Increasing the rate of vaccination with effective vaccines is important to mitigate the large toll of BRDC-related economic losses, however many challenges still remain in the use of these vaccines.

## 8. Vaccination Challenges

The development and deployment of effective vaccines against the key BRDC pathogens remains a challenge. Due to the multifaceted nature of BRDC, the most obvious challenge is the need to vaccinate against many different infectious agents. Whilst multivalent vaccines have been developed, there is still need for multiple vaccination regiments to be coordinated.

### 8.1. Lack of Evidence for Efficacy in the Field

Indeed, perhaps the most prominent issues of BRDC vaccination are determining the efficacy of available vaccines in the field (i.e., under natural conditions), and also the timing of administration. Whilst many studies testing the efficacy of BRDC vaccines have been undertaken under experimental conditions and virus challenge, there is a lack of evidence for the efficacy of vaccines against BRDC during natural conditions. Challenge studies, whilst important for determining the potential for a vaccine to be effective, have the disadvantages of an atypical environment and unnatural timings of pathogen exposure. Certain field trials have reported significantly lowered morbidity and mortality in vaccinated calves compared to non-vaccinated calves with multivalent vaccines against [133,134,135]. However, there is a lack of field trials investigating the prevention of BRDC for the many vaccines that have been developed. Additionally, while there are a number of experimental trials showing the protective effect of vaccinating young dairy calves with MLV vaccines [126,128,136,137], there is very limited evidence for the reduction of naturally occurring BRDC after vaccination of calves at this age (summarised in [125])

### 8.2. Importance of BRDC-Causing Pathogens Included in Vaccines 

It is also not entirely clear which components of the vaccines are the most important for calves to be vaccinated against in order to prevent BRDC. For example, a study comparing two multivalent vaccines (the first containing BoHV-1, types I and II BVDV, *M. haemolytica* and *P. multocida*), and the second containing BoHV-1, type 1 BVDV, BRSV, PI3V and *M. haemolytica*) found significantly lower BRDC mortality rates in the first vaccine group compared to the second [138]. This may suggest that inoculation against BoHV-1 and type II BVDV are more important in preventing BRDC than inoculation against BRSV and PI3V. However, no negative control group was included in this study. In addition to this omission of controls, differences in viral strains and adjuvants included in the two vaccines make it unclear precisely what differences are responsible for the variation in mortality rates [138]. Another study found no significant difference in mortality between calves that received a pentavalent (BoHV-1, BVDV types I and II, BRSV and PI3V) vs. a trivalent (BoHV-1 and BVDV types I and II) vaccine, again suggesting that the inclusion of BRSV and PI3V vaccination did not improve calf survival rates [139].

Given the fact that BRDC is induced by polymicrobial co-infections, it is imperative to vaccinate animals against multiple pathogens in order to provide sufficient protection. Studies examining respiratory co-infections have highlighted the increased severity of disease compared to infection with lone pathogens. This has been demonstrated for co-infections involving BVDV and *Pasteurella haemolytica* [140], BRSV and *H. somni* [102,141], and BRSV and *P. multocida* [24,142], among others. Specifically, BRSV infection of the lower respiratory tract has been shown to increase adherence of *P. multocida* which could contribute to the development of severe pneumonia [24,142]. Often, studies of calves suffering from respiratory disease detect infection with multiple viral and/or bacterial agents [143,144,145]. This highlights the importance and challenge of vaccinating calves against many potentially harmful pathogens in order to prevent BRDC.

Currently, the exact mechanisms in the development of BRDC remain unclear. It is conceivable that the inclusion of additional vaccines in the anti-BRDC inoculation routine could further reduce the incidence of BRDC. Additional pathogens implicated to play a role in BRDC include bovine adenoviruses [146,147], bovine coronavirus [147,148,149,150,151], bovine rhinitis A virus [13,151,152] and influenza C or D [13,151,153]. A metagenomics study found the top three viruses significantly associated with BRDC were bovine adenovirus 3, bovine rhinitis A virus and bovine influenza D virus [13]. In the case of influenza D, while it is known to cause mild disease, experimental infection of cattle leads to a significant increase of neutrophils in the trachea, which could predispose animals to BRDC [154]. Despite the associations with these additional viruses to BRDC, their roles in the development of the disease are not well understood and they are not included in the routine anti-BRDC vaccination regime. Further studies would be necessary to ascertain the impact of vaccination against these additional pathogens on BRDC incidence. One study has already demonstrated moderate protection of an influenza D virus vaccine against homologous challenge [155].

### 8.3. Timing of Vaccination and Maternal Antibodies

Deciding on the appropriate and most efficient time of vaccination is a further challenge to the BRDC vaccination effort. It is often thought that vaccination of neonatal calves is ineffective due to the presence of high amounts of maternal antibodies (MA) which can interfere with the development of immunological protection (reviewed in [156]), and this has been previously demonstrated [124,157,158,159]. However, other studies have shown effective vaccination in the presence of maternal antibodies using MLV vaccines [128,136,137,160]. One solution to the possible MA interference in immune stimulation is intranasal vaccination, or a primary intranasal vaccination to prime mucosal immunity, followed by a later parenteral vaccination boost. One study testing the prime-boost method found no difference in BRDC morbidity or mortality in recently weaned calves primed with an intranasal or injected multivalent vaccine [161]. However, a similar study did find that 2 month old calves primed at birth with an intranasal vaccine and then boosted with a multivalent inactivated vaccine developed higher levels of BRSV-specific neutralising antibodies compared to a group boosted with a similar MLV vaccine [162]. Overall, intranasal vaccines have not yet been shown to be any more effective in the prevention of BRDC compared to injected vaccines. Indeed, they have been shown to be inferior when inoculating beef calves at the age of weaning (reviewed in [125])

### 8.4. Timing of Vaccination and Immunological Stress

With the issue of vaccinating in the presence of MA, the timing of vaccination in the beef industry has shifted to vaccinate older calves after transport to feedlots. This solution however presents additional problems as calves are often vaccinated during stressful transition times. This means they are more likely to contract BRDC-causing infections when immune protection has not yet been established. One study found that vaccination of calves on arrival to feedlot resulted in an increased BRDC morbidity, mortality and poorer growth [163]. This could be due to the timing of vaccination, or perhaps that the calves were only vaccinated against viral components and not against the bacterial pathogens associated with BRDC.

Due to beef calves arriving at feedlots being considered high-risk for BRDC, investigations into delaying or bringing forward vaccination protocols have been performed. Overall, the evidence suggests that delaying vaccination in high-risk cattle is no more beneficial than vaccination on arrival [164,165,166], suggesting vaccination prior to arrival confers better protection [167]. Altering vaccination time avoids inoculation of animals when they may be experiencing stress-induced immunosuppression which may hinder the generation of a protective immune response. However, differences in vaccine efficacy in immunosuppressed animals has been observed depending on the type of vaccine administered. During a study in which cattle were acutely, chronically or not stressed, vaccination with a multivalent viral (MLV) and bacterial (inactivated) vaccine resulted in an increased antibody response specific for BoHV-1 and BVDV in the stressed animals, and a decreased anti-*M. haemolytica* antibody response in the same animals, compared to non-stressed controls [168]. This suggests that a suppressed immune system can actually allow increased replication of MLV vaccine elements, leading to a boosted immune response to vaccination. Whilst there may be safety concerns with such an enhanced response, it is clear that timing of anti-BRDC vaccinations not only depends on the age of the animal, but also the vaccine component.

### 8.5. Additional Issues

Further issues in the endeavour to produce an effective BRDC vaccination programme are mainly logistical. One such issue is the possible low adherence rates of herd owners to follow through with appropriate booster vaccinations in order to ensure full immune protection in calves. One survey in the UK in 2014 reported that only 48% of respondents had administered a second dose of vaccine within the recommended timeframe, and 14% had commenced vaccination before the recommended earliest age [169]. 

Appropriate storage of vaccines on farms may be another issue in achieving effective vaccination levels. One study involving 17 farms surveyed in South-West England found that all fridges monitored during the study had experienced internal temperatures outside the recommended storage temperature (between 2 and 8 °C) at least once over the 7 month monitoring period [170].

## 9. Conclusions and Necessary Developments

In summary, it is clear that current vaccination rates are not high enough to ensure prevention of BRDC and pneumonia in calves. Furthermore, there are a lack of vaccine trials in the field, with calves of different ages, showing prevention of naturally occurring BRDC by vaccination against various pathogens. It is important to decipher which vaccines (type, pathogen, and route of administration) are most effective under different conditions, taking into account the age of the animal and state of stress. The overall evidence suggests that calves are best vaccinated when young and healthy in order for immunity to be established prior to transportation, stressful changes in environment and potential pathogen exposure, which, as detailed above, can result in infection-induced immunosuppression. Further research is still necessary to determine which combination of vaccines will offer the best protection against BRDC from an early age.

## Figures and Tables

**Table 1:** Commercially available BRDC vaccines. An overview of the viral and bacterial vaccinations available as of February 2021.

Vaccine	Type	Component(s)
Bovalto Pastobov	Type A1 antigen	*M. haemolytica*
Rispoval Pasteurella	Inactivated	*M. haemolytica*
Rispoval RS	MLV	BRSV
Bovalto Respi Intranasal	MLV	BRSV PIV3
Imuresp RP	MLV	BoHV-1, PIV3
Rispoval 3	MLV	BRSV, PIV3, BVDV
Rispoval 4	MLV	BRSV, PIV3, BVDV, BoHV-1
Rispoval RS + PI3 Intranasal	MLV	BRSV, PIV3
Hiprabovis SOMNI/Lkt	Inactivated	*M. haemolytica* and *H. somni*
Bovalto Respi 3	Inactivated	*M. haemolytica* and PIV3
Bovilis Bovipast RSP	Inactivated	*M. haemolytica* and BRSV
Bovalto Respi 4	Inactivated	BRSV, PIV3, BVDV, *M. haemolytica*

## Data Availability

The data presented in this study are collected from the cited literature.
